# Quality of Ceftriaxone Sodium in Lyophilized Powder for Injection Evaluated by Clean, Fast, and Efficient Spectrophotometric Method

**DOI:** 10.1155/2017/7530242

**Published:** 2017-09-05

**Authors:** Patrícia Vidal de Aléssio, Ana Carolina Kogawa, Hérida Regina Nunes Salgado

**Affiliations:** Department of Pharmaceutics, School of Pharmaceutical Sciences of Araraquara, Universidade Estadual Paulista (UNESP), Araraquara, SP, Brazil

## Abstract

Ceftriaxone sodium, an antimicrobial agent that plays an important role in clinical practice, is successfully used to treat infections caused by most Gram-positive and Gram-negative organisms. Since there are few rapid analytical methods for ceftriaxone analysis to use in the pharmaceutical routine, the aim of this research was to develop a new method able to quantify this cephalosporin. Therefore, a sensitive, rapid, simple UV spectrophotometric method for the determination and quantification of ceftriaxone sodium was proposed. The UV detector was set at 241 nm. Beer's law obeyed the concentration range of 10–20 *µ*g mL^−1^. Statistical comparison of the results with a well-established reported method showed excellent agreement and proved that there is no significant difference in the accuracy and precision. Intra- and interday variability for the method were less than 2% relative standard deviation. The proposed method was applied to the determination of the examined drugs in pharmaceutical formulations and the results demonstrated that the method is equally accurate, precise, and reproducible as the official methods.

## 1. Introduction

Currently, analysis of pharmaceutical products is one of the most essential and promising approaches to enable the assurance the efficacy of drugs and medicines by pharmaceutical industries. In this way analysis of these matrixes is challenging due to their complex composition and unique characteristics.

In medicine there are several groups of antimicrobial agents; among them there is an important group that provides medical attention because of their efficacy in the treatment of infections caused by most Gram-positive and Gram-negative organisms, which are the cephalosporins [1–3].

**Figure 1:**
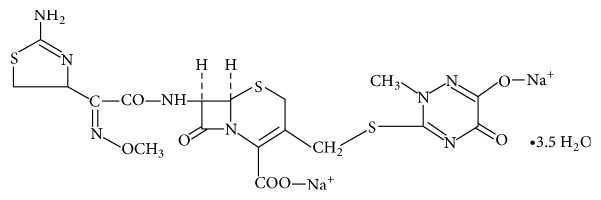
Chemical structure of ceftriaxone sodium (CAS 104376-79-6).

Cephalosporins are produced by having the key intermediate for semisynthetic production of a large number of cephalosporins which is 7-aminocephalosporanic acid, which is formed by hydrolysis of cephalosporin C produced by fermentation. Ceftriaxone sodium is semisynthetic cephalosporin of the third generation with high antibacterial activity, which is widely used in treatment of bacterial infections caused by susceptible, usually Gram-positive, organism, treatment of meningitis caused by aerobic Gram-negative bacteria, and other medical applications [4–8]. The chemical structure of ceftriaxone sodium is represented in Figure 1.

The literature describes some methods for the analysis of ceftriaxone sodium such as bioassay [9], HPLC [10–14], fluorimetry [15, 16], titrimetry [17], spectrophotometry [18–22], and micellar electrokinetic capillary chromatography [23–25].

However, there is still a need in the literature and in official compendia of fast, simple, and low cost method with concern on the health of the analyst and the environment to be applied in work routines in the quality control of pharmaceutical industries.

Considering its importance on the global scenario, the development of optimized analytical methods for the quality control of ceftriaxone is essential in seeking benefits for the human health and for the pharmaceutical companies.

The purpose of this work is to develop and validate a simple, fast, clean, sensitive, and accurate UV spectrophotometric procedure for determination of ceftriaxone sodium in injectable solution.

## 2. Experimental

### 2.1. Instrumentation

Absorbance measurements at 241 nm were carried out using UV Hewlett-Packard® KAYAK-XA spectrophotometer using quartz cells.

### 2.2. Materials and Reagents

All the chemicals used were of analytical grade. Ceftriaxone sodium (99.9%) was acquired by Sigma Aldrich® (St. Louis, USA) and the commercially available powders for injection were kindly supplied by União Química® Pharmaceutical Industry (Pouso Alegre, Brazil).

For the preparation of solutions, ultrapure water was obtained through a Milli-Q Plus purifier® (Millipore, USA).

- *Standard solutions of ceftriaxone sodium (reference standard):* they were prepared by dissolving 10.0 mg ceftriaxone sodium reference substance in ultrapure water in a 10 mL volumetric flask (1000 *µ*g mL^−1^).
- *Ceftriaxone sodium solutions*: aliquot volumes of standard stock solution containing 1000.0 *µ*g mL^−1^ reference substances were transferred to 25 mL volumetric flasks and diluted with ultrapure water to obtain suitable concentrations containing 10.0–20.0 *µ*g mL^−1^. The absorbance values of the resulting solutions were measured at 241 nm against reagent blank (ultrapure water). The test was performed in triplicate on three consecutive days.

Preliminary tests were carried out in order to get the best analytical conditions.

### 2.3. Method Validation

The method validation was performed according to the International Conference on Harmonisation (ICH) guidelines. The following validation characteristics were addressed: linearity, accuracy, precision, and robustness.

#### 2.3.1. Linearity

In order to assess the validity of the assay, 10 mg ceftriaxone sodium reference substance was dissolved in ultrapure water in 100 mL volumetric flask (1000.0 *µ*g mL^−1^). Appropriate aliquots of this solution were diluted with the water, yielding concentrations of 10.0, 12.0, 14.0, 16.0, 18.0, and 20.0 *µ*g mL^−1^. Triplicate preparations of each concentration were performed.

#### 2.3.2. Accuracy

The accuracy of the method was evaluated by addition of three different amounts of ceftriaxone sodium reference standard solution to sample solution. Recoveries were determined at three concentration levels being a low, medium, and high concentration by adding known amounts of reference substance to the sample, with the objective of verifying the accuracy of the proposed method. Aliquots of 0.5 mL of the sample stock solution (1000.0 *µ*g mL^−1^) were transferred to 50 mL volumetric flasks and aliquots were added of 0.64, 0.80, and 0.96 mL ceftriaxone sodium reference stock solution (1000.0 *µ*g mL^−1^), equivalent to 2.8, 6.0, and 9.2 *µ*g mL^−1^ of the ceftriaxone sodium reference substance. After this procedure, dilutions to volumes were made with the ultrapure water to give final concentrations of 80, 100, and 120% for R1, R2, and R3, respectively, of the sample concentrations used in the assay.

#### 2.3.3. Precision

Precision data for this validation were determined as recommended by ICH guidelines. The precision of the developed method was assessed in terms of repeatability and intermediate precision by analyzing six replicate quality control standard samples at 16 *µ*g mL^−1^.

#### 2.3.4. Robustness

Robustness can be established by changing the conditions of the proposed method, varying the wavelength of 239 nm and 243 nm. Each test was performed in triplicate. Robustness of the method was indicated by the general RSD% of the data in each condition variable.

## 3. Results and Discussion

Spectrophotometric analyses play an important role in quality control laboratories. These techniques have been successfully used some decades ago. However, with chromatographic advent, this efficient method was less used, even with its excellent results after its validation.

Ultraviolet and visible spectrophotometer has become a popular analytical instrument in the modern day laboratories and these techniques provide unique advantages which include availability, simplicity of operation, low cost, speed, precision, and accuracy, hence making them a powerful tool in chemical analysis [26–29].

After a literature search aiming to find a rapid method to quantify ceftriaxone during production phase in the pharmaceutical industry, the majority was HPLC, which use toxic mobile phase such as methanol and acetonitrile. For this reason, we aimed a validation of a rapid and ecological technique focusing in a no residue generation.

A useful spectrophotometric method for quantitation of ceftriaxone sodium was developed and validated by linearity, accuracy, precision, repeatability, and robustness. For drug analysis in quality control, the simplest and fastest procedures could be applied.

The absorption spectrum showed maximum absorption peak of 241 nm, using an aqueous solution of ceftriaxone sodium to 16 *µ*g mL^−1^. According to the Beer-Lambert law, the concentration range of 10.0 to 20 *µ*g mL^−1^ was linear, giving a correlation coefficient of 0.9992.

Accuracy is demonstrated by the % recovery and % RSD of 6 replicates of 12.8, 16.0, and 19.2 *µ*g mL^−1^ (R1, R2, and R3, resp.) solutions and 6 replicates of 16.0 *µ*g mL^−1^ solutions (Table 1).

The maximum RSD (%) was found to be 0.76, and the accuracy is shown by the agreement between 98.0 and 102.0% for the accepted value (the value was found to be 101.28% for powder for injection).

Results of intra- and interday precision checks showing variability as % RSD of the slopes of the calibration curves, when analyzed on the same day (*n* = 6) and on 3 consecutive days, were less than 2% (Table 2).

Robustness of the method was checked by deliberately altering one critical parameter by minor variations. The variation of the wavelength was not significantly different; the values obtained are presented in Table 3.

We could conclude that the proposed spectrophotometric method is simple, rapid, and with low reagent cost and can therefore be applied for the determination of ceftriaxone sodium in lyophilized powder for injection.

The proposed method has been successfully applied to the determination of ceftriaxone in pharmaceuticals and the results obtained are interchangeable with the results obtained by the HPLC method [30]. The applicability of the method was examined and results are highly reproducible; it can be used for routine analysis for quality control of ceftriaxone sodium in pharmaceutical industry.

## 4. Conclusions

The validated method met all the requirements recommended by the scientific literature for validation of analytical methods. Therefore, it is able to quantify ceftriaxone sodium in lyophilized powder for injectable solution, with the advantages of being a method of simple execution, of low analysis time, and that uses just purified water, an environmentally friendly solvent that presents low cost, easy disposal, and low toxicity.

Moreover, it can be used to monitor industrial process control and during quality control process. The method proved to be rapid, precise, and accurate and can be highly applicable in virtue of being inexpensive; the developed method does not involve any stringent reaction conditions, using water solvent. Therefore it could be used easily for the routine analysis of ceftriaxone sodium and their formulations.

## Figures and Tables

**Table 1:** Recovery test of ceftriaxone sodium powder for injection.

Analysis	Added (*µ*g mL^−1^)	Recovered (*µ*g mL^−1^)	Recovery (%)
R1	2.80	2.85	101.82
R2	6.00	6.07	101.22
R3	9.20	9.27	100.79

**Table 2:** Determination of the interday precision between analysts and for the UV spectrophotometric analysis of ceftriaxone sodium.

Interday					Among-analysts			
Sample	Day	Content^a^ (g/vial)	Content^a^ (%)	RSD^b^ (%)	Analysts	Content^a^ (g/vial)	Content^a^ (%)	RSD^b^ (%)
1	1	1.001	100.10	1.44	A	1.019	101.95	
	2	0.991	99.11		B	0.993	99.30	0.71
	3	1.019	101.95					

^a^Mean of six replicate analyses; ^b^RSD = relative standard deviation.

**Table 3:** Results of the evaluation of the robustness of the analytical method by UV spectrophotometry.

Sample	Wavelength (nm)	Content^a^ (g/vial)	Content^a^ (%)	RSD^b^ (%)
1	239	1.018	101.88	0.35
	241	1.018	101.80	
	243	1.015	101.59	

^a^Mean of six replicate analyses; ^b^RSD = relative standard deviation.
